# B Cell Development in the Bone Marrow Is Regulated by Homeostatic Feedback Exerted by Mature B Cells

**DOI:** 10.3389/fimmu.2016.00077

**Published:** 2016-03-22

**Authors:** Gitit Shahaf, Simona Zisman-Rozen, David Benhamou, Doron Melamed, Ramit Mehr

**Affiliations:** ^1^The Mina and Everard Goodman Faculty of Life Sciences, Bar-Ilan University, Ramat-Gan, Israel; ^2^Department of Immunology, Rappaport Faculty of Medicine, Technion – Israel Institute of Technology, Haifa, Israel

**Keywords:** B lymphocytes, BrdU, computer simulation, homeostatic feedback, mathematical modeling

## Abstract

Cellular homeostasis in the B cell compartment is strictly imposed to balance cell production and cell loss. However, it is not clear whether B cell development in the bone marrow is an autonomous process or subjected to regulation by the peripheral B cell compartment. To specifically address this question, we used mice transgenic for human CD20, where effective depletion of B lineage cells is obtained upon administration of mouse anti-human CD20 antibodies, in the absence of any effect on other cell lineages and/or tissues. We followed the kinetics of B cell return to equilibrium by BrdU labeling and flow cytometry and analyzed the resulting data by mathematical modeling. Labeling was much faster in depleted mice. Compared to control mice, B cell-depleted mice exhibited a higher proliferation rate in the pro-/pre-B compartment, and higher cell death and lower differentiation in the immature B cell compartment. We validated the first result by analysis of the expression of Ki67, the nuclear protein expressed in proliferating cells, and the second using Annexin V staining. Collectively, our results suggest that B lymphopoiesis is subjected to homeostatic feedback mechanisms imposed by mature B cells in the peripheral compartment.

## Introduction

B cell development starts in the bone marrow (BM) and continues in the spleen to final maturation. Developmental progression is guided by sequential events leading to assembly, expression, and signaling of the B cell antigen receptor (BCR). Heavy (H) and light (L) chain immunoglobulin genes are rearranged at the pro-B and pre-B stages (respectively), and complete surface IgM is expressed at the immature stage (1–5). Further developmental progression and maturation in the periphery are limited by positive and negative selection, mediated by BCR signaling and aiming to construct a non-self-reactive, immune-competent repertoire of naïve B cells (2, 6). In the adult mouse, the number of mature B cells remains constant, despite continuous production of new cells in the BM. It has been estimated that 1–2 × 10^7^ immature B cells are generated daily in the adult mouse and leave the BM as transitional B cells (7), but only about 3% enter the pool of mature B cells (8). Another source of B cell input to the peripheral compartment is antigen-driven proliferation. Cell death due to several factors including self-reactivity (9), incomplete maturation (10), competition for follicular niches (11), and trophic mediators (12) limits the size of the peripheral compartment. Thus, the balance between cell production and cell death maintains the size of the peripheral B cell compartment unchanged (13).

To understand this homeostatic control, it is of critical importance to quantify the rate of B lymphopoiesis under physiological conditions. This question has been the subject of several investigations in the past, employing different approaches. Experiments based on an irradiation/autoreconstitution model of marrow-derived B cell differentiation demonstrated that frequencies of pro-/pre-B cells (14, 15) and immature B cells (16) increase after irradiation, and so does differentiation and maturation (17, 18). It has also been demonstrated that upon immunological challenge, BM B lymphopoiesis increases, as measured by cell number, cell division, and turnover (19, 20). These experiments supported the concept that B lymphopoiesis is regulated by a feedback mechanism imposed by peripheral B cells and/or environmental stimuli. Other experiments have used mixed BM chimeras to suggest that B cell production in the BM is not subjected to feedback inhibition by the peripheral B cell compartment but is rather autonomously regulated (21, 22). Similarly, in mice depleted of B cells from birth by anti-IgM antibodies, or in hematologically mutant mice (20), no difference was found in the proliferation rate of pre-B cells relative to controls. There are also contradicting results about the influence of serum immunoglobulins on the regulation of developing B cell populations in the BM (23, 24). Possible causes for the different conclusions may be attributed to the profound differences in the experimental systems used to eliminate the peripheral B cell compartment and to the complexity of both models in affecting other lineages and tissues. Moreover, quantification of B cell production in each of the above studies was based on a single measurement of cell frequency, which may be differentially affected in each experimental setup, rather than on a series of kinetic measurements. Thus, while the concept that B cell homeostasis is mediated through a feedback effect exerted by peripheral B cells on developing B cells has been proposed earlier (14, 15), it has not yet been proven. In order to address this question, we have used the human CD20Tg mouse model, where peripheral B cells can be specifically eliminated using anti-hCD20 antibodies with no residual effect on other cell populations (25), in a study combining BrdU labeling over time and mathematical modeling.

Mathematical models are often used to extract parameters from labeling experiments for better interpretations of the results, which otherwise are very often misinterpreted (26, 27). We have previously used the combination of BrdU labeling and mathematical models to study the development and maturation of B cells (28–30). These studies led to the discoveries of phenotypic reflux in B cell development (28) and the role of the Transitional-3 compartment as an anergic pool accounting for most follicular mature B cell death in WT mice (30) showed that, in contrast, in Lyn^−/−^ mice, the less stable follicular B cells died directly (31).

In the present study, we asked whether or not the rate of B lymphopoiesis is increased upon demand for peripheral B cells. To address this issue, we expanded our previous mathematical model to include the mature recirculating subpopulation in the BM and fit the model to data from BrdU labeling experiments on B cell-depleted and control mice. We report here that the rapid reconstitution of marrow B cell numbers after short depletion is due to increased proliferation in the pro-/pre-B cell compartment. BM subpopulations were reconstituted faster than subpopulations that develop in the spleen. The depletion also strongly affected the immature B cell subset; we found accelerated immature B cell death and lower rates of differentiation to transitional B cells in depleted mice compared to control mice. Finally, our new mathematical model, which includes the subset of mature recirculating B cells in the BM, predicts that the most probable source of mature B cells in the BM is inflow from the periphery to the BM, rather than from developing B cells.

## Materials and Methods

### Mice and B Cell Depletion

For these experiments, we used Balb/c mice that are normal or transgenic for human CD20 (hCD20 Tg) (25) at 3–4 months of age. The mice were housed and bred at the animal facility of the Faculty of Medicine, Technion, and all studies were approved by the institute’s committee for the supervision of animal experiments. To deplete B cells *in vivo*, hCD20Tg mice were injected intraperitoneally with purified monoclonal mouse anti-hCD20 (clone 2H7) antibodies at 1 mg/mouse as described (25). Depletion was confirmed by flow cytometry analysis of peripheral blood cells. Mice were analyzed 34 days after depletion, when reconstitution of peripheral compartment reached 50–60% (32).

### Antibodies and Flow Cytometry Analysis

Isolation, staining, and flow cytometry analysis for B cell populations in BM and spleen were carried out as described earlier (32–34). Briefly, single-cell suspensions from BM and spleen were stained for surface marker expression using PE-, allophycocyanin-, PerCP-, and biotin-conjugated Abs, visualized with Streptavidin Brilliant Violet 421 (BioLegend). Antibodies used for cell staining were CDR45R/B220 RA3-6B2 (BioLegend), CD23 clone 2G8 [Southern Biotechnology Associates (SBA)], CD43 clone S7 (BD Biosciences), CD93 (AA4.1) (eBioScience), human CD20 (hCD20) clone 2H7 (eBioscience), and IgM (Jackson ImmunoResearch). Stained cells were analyzed by a fluorescence-activated cell sorter (FACS) with viable lymphocyte gate as defined by forward and side light scatter. Data for five-color analysis were collected on a FACSCyAn ADP (Beckman Coulter Inc.) and were analyzed using the FlowJo software (Tree Star). B cell subsets were defined using the following surface markers: in the BM: pro-/pre-B cells (B220^+^/AA4.1^−^/IgM^−^), immature (B220^+^/AA4.1^+^/IgM^+^), and mature-circulated (B220^+^/AA4.1^−^/IgM^+^); in the spleen: transitional (B220^+^/AA4.1^+^/IgM^+^) and mature (B220^+^/AA4.1^−^/IgM^+^), as described in Ref. (32–36).

### Analysis of BrdU Incorporation

*In vivo* BrdU incorporation was performed as described (28). Briefly, mice were intraperitoneally injected with two doses per day of 2 mg/mouse of BrdU. On days 2, 4, and 7, BM and spleen cells were isolated and stained with fluorescently labeled antibodies for surface markers, as detailed in the previous section. Subsequently, cells were stained with a FITC-conjugated anti-BrdU antibody using the BrdU Flow Kit (BD Biosciences), according to the manufacturer’s protocol.

### Analysis of Ki67 Expression

B cell proliferation was estimated in control and B cell-depleted mice 21 days after B cell depletion by flow cytometry using an intracellular antibody against Ki67, the nuclear protein expressed in proliferating cells, as described in Ref. (37). For determination of Ki67-positive cells, cells were first stained for surface B cells markers: B220, AA4.1, and IgM, followed by intracellular staining for Ki67 as follows. Cells were fixed and permeabilized in Cytofix/Cytoperm solution (BD Biosciences) for 20 min at 4°C and then incubated with Ki67 Alexa647-conjugated monoclonal antibody (Santa Cruz Biotechnology).

### Analysis of Apoptosis by Annexin V

The extent of apoptosis in developing B cells was determined by Annexin V staining in control and B cell-depleted mice 21 days after B cell depletion, as described in Ref. (36). BM cells were stained for B220, AA4.1, and IgM, followed by Annexin V (Biolegend, catalog number 640920) according to the manufacturer’s protocol. Cells were then analyzed by flow cytometry.

### Statistical Analysis of Experimental Data

We first tested whether there are significant differences in BrdU labeling kinetics between the control and the depleted mice using generalized linear model (GLM) repeated measures, a method based on analysis of variance (ANOVA). Repeated measures ANOVA is the equivalent of the one-way ANOVA, but is used for related rather than independent measurements, and is the extension of the dependent *T*-test. The test detects any overall differences between related means. The dependent variables should be continuous and the independent variables categorical. Each dependent variable is represented by as many variables as there are measurement times (e.g., Imm_2, Imm_4, and Imm_7 are the BrdU fractions of labeled immature cells after 2, 4, and 7 days). Mouse type is the categorical predictor variable. The analysis was performed using the SPSS software.

### Mathematical Models of B Cell Subpopulations in the Spleen

**Figure 1:**
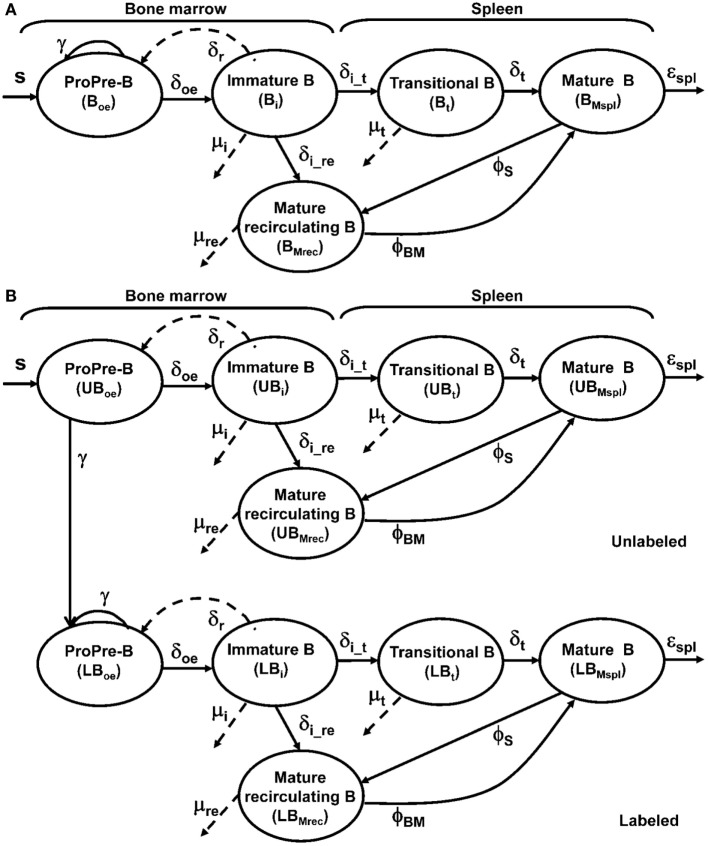
**Model of B cell population dynamics in the bone marrow and spleen**. **(A)** The model of maturing B-cell population dynamics in the bone marrow and spleen is shown. All population processes – differentiation, proliferation, and death – are described by arrows. The rate of each process is given near the corresponding arrow. S is the source of B lineage precursors (cells/6 h), parameters δ denote differentiation rates, μ mortality rates, γ proliferation rates, and ϕ flow rates. **(B)** The model for labeling dynamics of developing B cells in the bone marrow assumes that cells in the unlabeled pro-/pre-B cell compartment move to the corresponding labeled compartment upon dividing. The model also assumes that each labeled cell remains labeled for the duration of the experiment.

**Figure 2:**
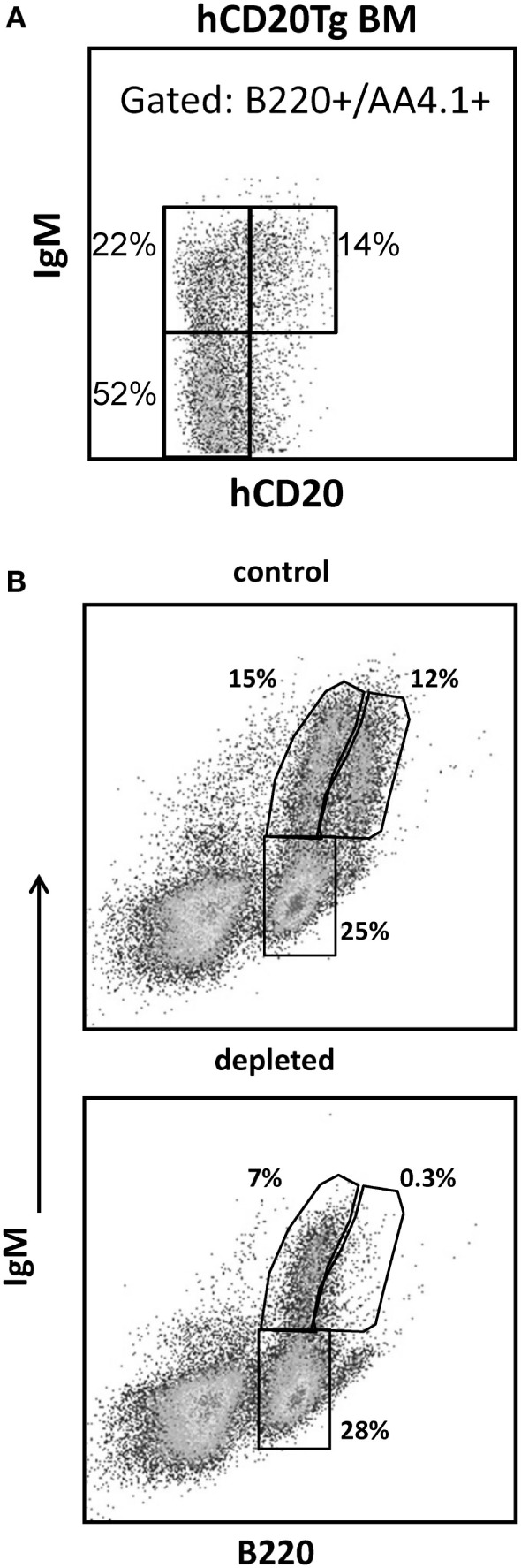
**Selective depletion of BM B cell subsets in an hCD20Tg mouse**. **(A)** BM cells from hCD20Tg mouse were stained for B220, IgM, AA4.1, and hCD20. For analysis, viable lymphocyte were defined by forward and side light scatter, and gates were set to analyze B220^+^/AA4.1^+^ early developing B cells (pro-/pre-B and immature B cells) and to exclude circulated B220^+^/AA4.1^−^ and non-B lineage cells. Gated cells were analyzed for expression of IgM and hCD20. **(B)** hCD20Tg mice were injected with anti-hCD20 antibodies. Three days after injection, BM cells were stained for B220 and IgM and analyzed for the following B cell subsets: pre-/pro- (B220^+^/IgM^−^), immature (B220^+^/IgM^+^), and circulated mature (B220hi/IgM^+^) B cells, relative to control hCD20Tg mice that were not treated. The results shown are representative of three to four mice in each group.

We fit the BrdU data from control and B cell-depleted mice to our mathematical models of B cell development in the BM and maturation in the spleen. The model (Figure 1A) is fully described by Eqs 1–5 below and has been constructed as follows. First, for each population, we assigned a variable denoting the number of cells in each population. The current data did not include pro- and pre-B subpopulations; hence, we decided to include in the model a population that combined both subsets, *B*_oe_, to represent the cells in the pro- and pre-B cell subsets that eventually differentiate to the immature subset (henceforth called pro-/pre-B cells). Transitional B cells are modeled here as one subset, ignoring their division into several maturation stages, because the focus of this study is on B cell development in the BM. The current data include the mature recirculating B cells subset in the BM, so we added this subset to our mathematical model (Figure 1A; Eq. 3). The numbers of cells in the immature, mature recirculating, transitional, and splenic mature subsets are represented by the variables *B*_i_, *B*_Mrec_, *B*_t_, and *B*_Mspl_, respectively (Figure 2; Eqs 2–5).

Second, for each process in the model (that is, an arrow in Figure 1), a rate parameter was assigned; parameters were denoted by Greek letters to distinguish them from variables. For example, *B*_oe_ cells differentiate into the immature subset with a constant rate denoted by δ_oe_ (per 6 h, which is our simulation time unit). Third, the change in each population in each time unit (approximated by the derivative of the corresponding variable representing the number of the cells in this population) is given by a differential equation. The general form of each differential equation is of the form:

$$\frac{\text{d }\left(\text{variable}\right)}{\text{dx}}=\text{entry}+\text{proliferation}-\text{death}-\text{differentiation}$$

where each of the four right-hand terms is either a constant or a function of the variables and parameters. For example, the *B*_oe_ subpopulation is renewed from previous subsets at the constant rate *S*. This subpopulation proliferates after passing heavy or light chain rearrangements with a maximum rate γ (Figure 1A; Eq. 1). The proliferation term in Eq. 1 is the product of the number of cells that are able to proliferate (*B*_oe_), the maximum proliferation rate (γ), and a so-called “logistic” limit on the proliferation, which is a function that decreases with the available space for this population and is constructed as follows. The mature recirculating subpopulation includes mature recirculating B cells that compete with pro-/pre-B cells for survival niches (38), as do plasma cells. Therefore, the carrying capacity parameter, *K*, that limits the proliferation rate of the pro-/pre-B cell subpopulation includes the total number of cells in both the pro-/pre-B and the mature recirculating compartments (Eqs 1, 6, and 11), and the logistic term is thus $\left[1-\frac{\left(B_{\text{oe}}+B_{\text{Mrec}}\right)}{K}\right]$.

The mature recirculating B cell subpopulation in this model represents all the mature B cell subsets that re-enter into the BM from the periphery. Other feedback onto the pro-/pre-B cell division by any other cells, such as plasma cells, was not explicitly modeled, since those cells were not measured; however, their effect is implicitly modeled in part *via* the carrying capacity *K*, such that a decrease in *K* would be interpreted as competition by other cells for survival niches. Additionally, since the labeling data did not include pro- and pre-B cell subpopulations, a regulation of the source of pro-/pre-B cells by peripheral B cells or by other cells in response to the depletion was not explicitly examined (see Discussion).

Immature B cells either differentiate to BM mature cells at rate δ_i_re_, or emigrate from the BM to the spleen and differentiate to transitional B cells at rate δ_i_t_ (Eqs 2–4). Transitional B cells differentiate to splenic mature B cells at rate δ_t_ (Eqs 4 and 5). After their maturation, splenic mature B cells can go back to the PB and then to the mature recirculating population in the BM. The flow of mature B cells from the spleen to the mature (recirculating) population in the BM is represented by the parameter ϕ_S_. The flow in the opposite direction is represented by the parameter ϕ_BM_ (Eqs 3 and 5).

**Figure 3:**
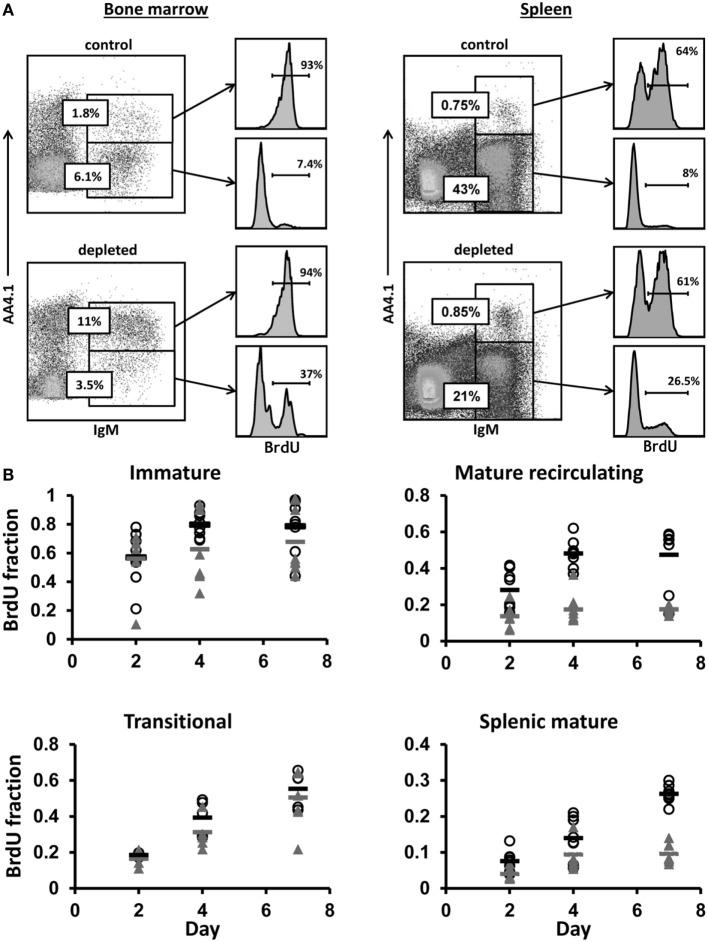
**Control and hCD20-depleted mice (34 days after depletion) were injected with BrdU and analyzed for BrdU labeling as detailed in Section “Materials and Methods.”** **(A)** BM and spleen cells from the control and the depleted mice were stained for IgM, AA4.1 and BrdU labeling. For analysis, viable lymphocytes were defined by forward and side light scatter, and the relative BrdU labeling in BM immature (IgM^+^/AA4.1^+^) and mature-circulated (IgM^+^/AA4.1^−^), and in spleen transitional (IgM^+^/AA4.1^+^) and mature (IgM^+^/AA4.1^−^) B cells was determined using gates set as shown (representative example). **(B)** BrdU labeling kinetics in B cell-depleted (black, open circles for each single mouse and band for the mean value) and control (gray, filled triangles for each single mouse and band for the mean value) mice. For total cell numbers, see Figure 4.

The death rates are denoted by μ_i_, μ_t_, and μ_rec_ for *B*_i_, *B*_t_, and *B*_Mrec_, respectively. The exit from the splenic mature B cell, including death or transition to other organs, is denoted by *ε*_spl._ Based on previous studies, it is assumed that the transitional subpopulation is not cycling (29, 39). We did not include the possibility that peripheral B cell division is also a source of the reconstitution, since a preliminary Ki67 staining showed no evidence for cell division among mature splenic B cells in either the depleted or control mice (data not shown). Thus, the differences in the BrdU labeling kinetics between control and depleted mice (Figure 3B) must be ascribed to differences in the entry-labeled cells from previous developmental stages.

**Figure 4:**
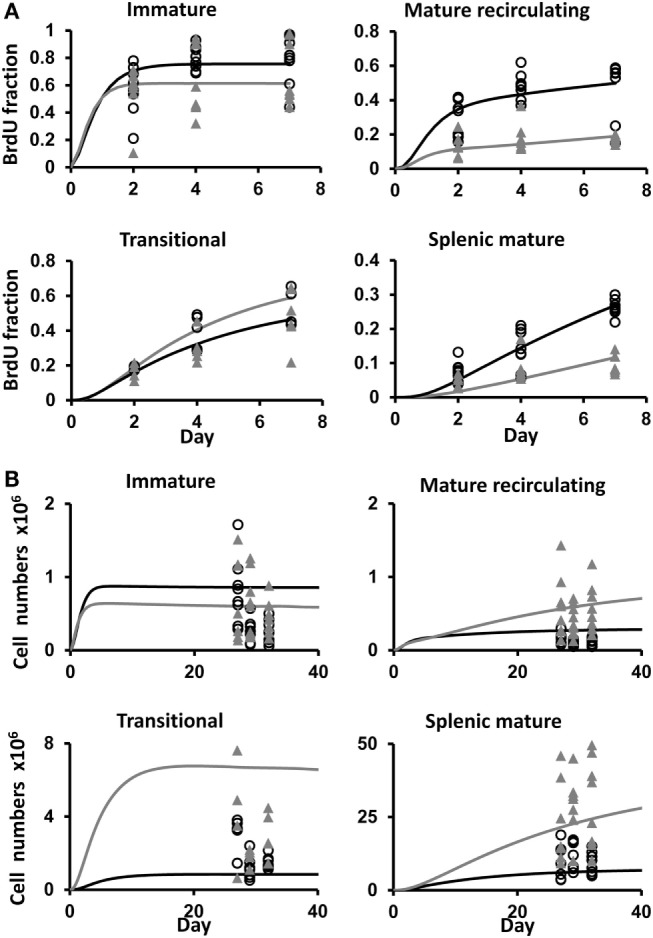
**(A)** BrdU labeling kinetics obtained by a simulation of the B cell development model using the parameter value set that gave the best fit to the data, shown (as lines) along with the data (symbols), for control (filled gray triangles and gray lines), and depleted mice (open black circles and black lines), in the immature, mature recirculating, transitional, and splenic mature B cell subsets. The *x*-axis shows the time from the start of labeling. **(B)** The ranges of total cell numbers in each B cell subset in the experimental measurements of control and depleted mice and the corresponding simulations [symbols and lines are as in **(A)**]. The *x*-axis shows the time from the start of B cell depletion. The parameter values used in these simulations are given in Table 1.

Thus, the model is completely described by the following equations.

(1) $$\begin{array}{l} \frac{\text{d}B_{\text{oe}}}{\text{dt}}=s+\left\{\gamma \left[1-\frac{\left(B_{\text{oe}}+B_{\text{Mrec}}\right)}{K}\right]-\delta_{\text{oe}}\right\}B_{\text{oe}}+\delta_{\text{r}}B_{\text{i}} \end{array}$$

(2) $$\begin{array}{l} \frac{\text{d}B_{\text{i}}}{\text{dt}}=\delta_{\text{oe}}B_{\text{oe}}-B_{\text{i}}\left(\mu_{\text{i}}+\delta_{\text{i\_t}}+\delta_{\text{r}}+\delta_{\text{i\_re}}\right) \end{array}$$

(3) $$\begin{array}{l} \frac{\text{d}B_{\text{Mrec}}}{\text{dt}}=\delta_{\text{i\_re}}B_{\text{i}}+\varphi_{\text{s}}B_{\text{Mspl}}-\left(\mu_{\text{re}}+\varphi_{\text{BM}}\right)B_{\text{Mrec}} \end{array}$$

(4) $$\begin{array}{l} \frac{\text{d}B_{\text{t}}}{\text{dt}}=\delta_{\text{i\_t}}B_{\text{i}}-B_{\text{t}}\left(\mu_{\text{t}}+\delta_{\text{t}}\right) \end{array}$$

(5) $$\begin{array}{l} \frac{\text{d}B_{\text{Mspl}}}{\text{dt}}=\delta_{\text{t}}B_{\text{t}}+\varphi_{\text{BM}}B_{\text{Mrec}}-\left(\varphi_{\text{s}}+\varepsilon_{\text{spl}}\right)B_{\text{Mspl}} \end{array}$$

### Modeling BrdU Labeling

The modeled subsets were divided into labeled and unlabeled B cell populations, and the dynamics of these subsets are given in Eqs 6–15 below. UB_oe_, UB_i_, UB_t_, UB_Mrec_, and UB_Mspl_ denote the numbers of unlabeled pro- and pre-, immature, transitional, BM recirculating mature, and splenic mature B cells, respectively (Eqs 6–10), and LB_oe_, LB_i_, LB_t_, LB_Mrec_, and LB_Mspl_ similarly denote the labeled subsets (Eqs 11–15). In these equations, *B*_oe_ = UB_oe_ + LB_oe_, *B*_i_ = UB_i_ + LB_i_, *B*_t_ = UB_t_ + LB_t_, *B*_Mrec_ = UB_Mrec_ + LB_Mrec_, and *B*_Mspl_ = UB_Mspl_ + LB_Mspl_. Cells in each unlabeled compartment move to the corresponding labeled subset upon dividing (Figure 1B). When an unlabeled cell divides during the labeling period, it leaves the unlabeled subpopulation. Its two daughter cells both become labeled cells and join the labeled subpopulation; hence, the number of unlabeled proliferating cells entering the labeled subset is multiplied by a factor of 2. We neglect BrdU toxicity (and thus assign the same death rates to labeled and unlabeled cells), as in previous work (28–31, 40); we found that when the BrdU experiment is very short – as in this case, only 7 days – the latter assumption holds. We also neglect labeling of the source cells, as in previous work (28).

(6) $$\begin{array}{l} \frac{\text{dU}{\text{B}}_{\text{oe}}}{\text{dt}}=s-\left\{\gamma \left[1-\frac{\left(B_{\text{oe}}+B_{\text{Mrec}}\right)}{K}\right]-\delta_{\text{oe}}\right\}\text{U}{\text{B}}_{\text{oe}}+\delta_{\text{r}}\text{U}{\text{B}}_{\text{i}} \end{array}$$

(7) $$\begin{array}{l} \frac{\text{dU}{\text{B}}_{\text{i}}}{\text{dt}}=\delta_{\text{oe}}\text{U}{\text{B}}_{\text{oe}}-(\mu_{\text{i}}+\delta_{\text{i\_t}}+\delta_{\text{r}}+\delta_{\text{i\_re}}\text{)U}{\text{B}}_{\text{i}} \end{array}$$

(8) $$\begin{array}{l} \frac{\text{dU}{\text{B}}_{\text{Mrec}}}{\text{dt}}=\delta_{\text{i\_re}}\text{U}{\text{B}}_{\text{i}}+\varphi_{\text{s}}\text{U}{\text{B}}_{\text{Mspl}}-(\mu_{\text{re}}+\varphi_{\text{BM}}\text{)U}{\text{B}}_{\text{Mrec}} \end{array}$$

(9) $$\begin{array}{l} \frac{\text{dU}{\text{B}}_{\text{t}}}{\text{dt}}=\delta_{\text{i\_t}}\text{U}{\text{B}}_{\text{i}}-\left(\mu_{\text{t}}+\delta_{\text{t}}\right)\text{U}{\text{B}}_{\text{t}} \end{array}$$

(10) $$\begin{array}{l} \frac{\text{dU}{\text{B}}_{\text{Mspl}}}{\text{dt}}=\delta_{\text{t}}\text{U}{\text{B}}_{\text{t}}+\varphi_{\text{BM}}\text{U}{\text{B}}_{\text{Mrec}}-(\varphi_{\text{s}}+\varepsilon_{\text{spl}}\text{)U}{\text{B}}_{\text{Mspl}} \end{array}$$

(11) dLBoedt=(2×Boe+LBoe) γ 1−(Boe+BMrec)K−δoeLBoe+δrLBi

(12) $$\begin{array}{l} \frac{\text{dL}{\text{B}}_{\text{i}}}{\text{dt}}=\delta_{\text{oe}}\text{L}{\text{B}}_{\text{oe}}-\left(\mu_{\text{i}}+\delta_{\text{i\_t}}+\delta_{\text{r}}+\delta_{\text{i\_re}}\right)\text{L}{\text{B}}_{\text{i}} \end{array}$$

(13) $$\begin{array}{ll} \frac{\text{dL}{\text{B}}_{\text{Mrec}}}{\text{dt}}= & \;\delta_{\text{i\_re}}\text{L}{\text{B}}_{\text{i}}+\varphi_{\text{s}}\text{L}{\text{B}}_{\text{Mspl}}-\left(\mu_{\text{re}}+\varphi_{\text{BM}}\right)\text{L}{\text{B}}_{\text{Mrec}} \end{array}$$

(14) $$\begin{array}{l} \frac{\text{dL}{\text{B}}_{\text{t}}}{\text{dt}}=\delta_{\text{i\_t}}\text{L}{\text{B}}_{\text{i}}-\left(\mu_{\text{t}}+\delta_{\text{t}}\right)\text{L}{\text{B}}_{\text{t}} \end{array}$$

(15) $$\begin{array}{l} \frac{\text{dL}{\text{B}}_{\text{Mspl}}}{\text{dt}}=\delta_{\text{t}}\text{L}{\text{B}}_{\text{t}}+\varphi_{\text{BM}}\text{L}{\text{B}}_{\text{Mrec}}-\left(\varphi_{\text{s}}+\varepsilon_{\text{spl}}\right)\text{L}{\text{B}}_{\text{Mspl}} \end{array}$$

### Model Simulation Program Description

The mathematical models were simulated and fitted to the experimental data using a Matlab program. The program receives as input the experimental data and the ranges of parameter values within which the model should be run. In the simulation, we tested only parameter combinations that gave total cell numbers at steady state in each B cell subpopulation within a range of total cells numbers measured experimentally. Since the BrdU incorporation experiment in depleted mice was done before B cells were fully reconstituted, the cell populations have not yet reached a steady state; hence, we used control mouse total cell numbers to test parameter value sets for both mouse types, assuming that in the depleted mice, after full reconstitution, the total cell numbers would reach the same range as in the control mice.

For each parameter value set, the program integrates the model equations as follows. The initial conditions are 0 cells in all populations; labeling only starts after the populations have reached a steady state in the control mice, and 34 days after depletion in the depletion mice, as done in the experiments. For all runs, the program calculates the value of the fit of the model to the data (see below) and outputs the parameter values, the total cell numbers in each subpopulation, and the value of the fit. This process was performed for each type of mice (depleted and control).

### Model Parameter Estimation

For the purpose of finding parameter ranges, we used a Monte Carlo class algorithm for sampling named the Latin hypercube sampling (LHS) (41). LHS allows an unbiased estimate of the average model output, with the advantage that it requires fewer samples than simple random sampling to achieve the same accuracy. In LHS, each parameter range is divided into *N* equal probability intervals, and each interval is sampled exactly once, and thus, *N* values are tested for each parameter. *N* parameter combinations are set by assigning a value for each parameter, selected from a random bin. Using LHS allows us to run the simulation for a magnitude smaller number of combinations (41).

We used maximum likelihood parameter estimation (MLE) to determine the parameter values that maximize the probability [likelihood (L)] of the data. The best fit parameter value set is defined as the set of parameter values that yields the MLE for all time points and for all experimental repetitions (mice).

### Uncertainty Analysis

Input parameters for most mathematical models are not always known with a sufficient degree of certainty because of natural variation, error in measurements, or simply a lack of current techniques to measure them. The purpose of uncertainty analysis is to quantify the degree of confidence in the existing experimental data and parameter estimates. We furthermore used the profile likelihood method to obtain confidence intervals (CI) for all the mathematical model parameters (42–44). The likelihood ratio test statistic (*G*^2^) of the hypothesis *H*_0_: θ = θ0 (where θ0 is a fixed value of a specific parameter) equals the drop in 2lnL between the “full” model and the reduced model with θ fixed at θ0, i.e., *G*^2^ = 2[lnL(“Best fit”) − lnL(θ0)]. A 95% CI consists of those values of θ0 for which the test is not significant at a significance level 0.05; this is the case when *G*^2^ does not exceed 3.84 [the 95th percentile of the χ^2^(1) distribution]. In order to compare parameter values between mouse types, we examined the parameter’s 95% CI values. Very similar results were obtained when we used an Akaike’s information criterion (AIC)-based method, as in Ref. (30, 31, 34), indicating that these two methods for deriving parameter CI are equivalent.

### Sensitivity Analysis

Sensitivity analysis (SA) is another method for quantifying uncertainty in any type of complex model. The objective of SA is to identify critical inputs (parameters and initial conditions) of a model and to quantify the extent to which input uncertainty impacts model outcome(s). Linear regression analysis is a global SA technique, which is implemented by the Monte Carlo simulations. The linear regression analysis searched for linear trends of the total numbers of cells in each subpopulation, which are the model’s dependent variables, as function of the input parameters. Two linear relationship measures are examined: the Pearson correlation coefficient (*R*) and the partial correlation coefficients (partial *R* or PRCCs). PRCCs appear to be, in general, the most efficient and reliable among sampling-based indexes (45). PRCCs provide a measure of monotonicity after removal of the linear effects of all but one parameter. Here, we used a stepwise regression method in order to decide which of the input parameters are critical to the model outcome(s). The parameters were included in the regression analysis according to the change in the Pearson correlation coefficient.

## Results

### Faster BrdU Labeling of Mature B Cells in Depleted Mice Compared with Controls

To determine whether B lymphopoiesis in the BM is regulated by the size of the peripheral B cell compartment, we used hCD20Tg mice where hCD20 is expressed exclusively on B lineage cells, and administration of anti-hCD20 antibodies has been shown to deplete the peripheral B cells, leaving all other cell lineages intact (25). However, analysis of hCD20 expression during B cell development revealed that hCD20 expression in these mice starts only at the immature stage (IgM^+^), where about 40% of the cells in this population, mostly late immature (as revealed by high expression of IgM), express hCD20 (Figure 2A). Thus, about 36% of the B220^+^/AA4.1^+^ developing B cells are immature IgM^+^ cells, but only about 40% of them express hCD20 (14%, Figure 2A) and can be targeted for depletion upon administration of anti-hCD20 antibodies. The pro-/pre-B cells in these mice do not express hCD20 (Figure 2A). In agreement with this, administration of anti-hCD20 antibodies leads in the BM to the rapid elimination of the hCD20^+^ subgroup of immature B cells as well as circulated mature B cells but leaves the pro-/pre-B cell compartment unchanged (Figure 2B). Thus, BM analysis 3 days after treatment with anti-hCD20 antibodies revealed complete depletion of mature-circulated B cells (B220hi/IgM^+^). Owing to the antibody-mediated elimination of the hCD20^+^ cells in the immature B cell compartment (Figure 2A), in the current study, there was a reduction of about 50% in this compartment (B220^+^/IgM^+^, from 15 to 7%, Figure 2B); this reflects a similar reduction in cell numbers, since immature cell numbers in the depleted mice did not significantly differ from those in control mice (see below). There was no change in the size of the pro-/pre-B cell compartment, which does not express hCD20; in both control and depleted mice, it consisted of about 25% of B cells (Figure 2B). We conclude that this selective depletion enables the hCD20Tg mouse model to be used as an efficient model to test whether B lymphopoiesis is homeostatically regulated in the most physiological setup.

To explore whether B lymphopoiesis is homeostatically regulated, hCD20Tg mice were treated with anti-hCD20 antibodies to deplete B cells, thus generating conditions of peripheral “demand” for B cells. Thirty-four days after depletion [when reconstitution reached 50–60% (32)], we performed continuous *in vivo* BrdU labeling of the depleted and control mice for 7 days, and analyzed B cell subsets on days 2, 4, and 7 (*n* = 8 mice per time point). In the BM, we defined immature B cells as IgM^+^/AA4.1^+^ and mature-circulated B cells as IgM^+^/AA4.1^−^, and in the spleen, we defined transitional B cells as IgM^+^/AA4.1^+^ and mature B cells as IgM^+^/AA4.1^−^ (Figure 3A). Gates were set for each subset (Figure 3A), and the relative BrdU labeling in each subset was determined (Figure 3B).

No differences between control and depleted mice were seen in the immature (*p* = 0.391) and transitional B cell (*p* = 0.152) BrdU labeling kinetics (Figure 3B). However, there were significant differences in mature recirculating B cell labeling (*p* = 3.476E−007). The fractions of labeled mature recirculating B cells in depleted mice reached 60% by day 7, compared with 20% in the control mice. Similar significant differences between control and depleted mice were observed in the splenic mature B cell labeling kinetics starting at day 4 (*p* = 1.806E−007), when the fractions of labeled splenic mature B cells in depleted mice reached 25%, compared with 10% in the control mice.

### Higher Pro-/Pre-B Cell Proliferation in Depleted Mice

We fitted our mathematical model of B cell BM development and splenic maturation dynamics to the BrdU labeling data, and compared the best fit parameter values and 95% CI (obtained using the profile likelihood method) for the two mouse types (Table 1). Parameter ranges that show no overlap between the two mouse types – and hence are significantly different – are highlighted in Table 1. Figure 4A shows the model’s fit to the data with the best fit parameter value sets. The simulated total cell numbers at each stage of development of control and depleted mice were within the experimentally observed ranges (Figure 4B).

**Table 1:** **The 95% CI for parameter values obtained in simulations of labeling kinetics of control and depleted B cells**.

Parameter	Control		Depleted	
	Best fit	95% CI	Best fit	95% CI
γ	0.3	0.20–0.35	0.38	0.30–0.4
δ_oe_	0.5	0.32–0.55	0.25	0.20–0.48
μ_i_	0.10	0.02–0.2	0.53	0.2–0.75
δ_i_t_	0.60	0.50–0.75	0.20	0.05–0.50
δ_i_re_	0.19	0.10–0.22	0.158	0.10–0.20
Immature residence time	1.13	1.1–1.5	1.13	1.1–1.6
μ_t_	0.03	0.02–0.05	0.06	0.04–0.06
δ_t_	0.03	0.014–0.04	0.005	0.005–0.04
Transitional residence time	17.4	13.2–20.4	15.9	13.3–20.5
ϕ_BM_	0.94	0.6–10.0	0.95	0.7–10.0
μ_rec_	0.008	0.000–0.048	0.040	0.005–0.060
ϕ_s_	0.03	0.015–0.030	0.019	0.014–0.024
*ε*_spl_	0.008	0.008–0.009	0.018	0.014–0.022

*All parameters are given in units of 1/6 h, and residence times are given in units of 6 h. Cases where the ranges for control and depleted mice do not overlap are highlighted. Some of the parameter values were found to be very similar in the control and depleted cells, so we fixed them to constant values in the final series of runs. These constant values were *S* = 3 × 10^5^ cells/6 h, *K* = 6 × 10^6^ cells, and δ_r_ = 0.05, with all rates given as fraction per 6 h*.

**Figure 5:**
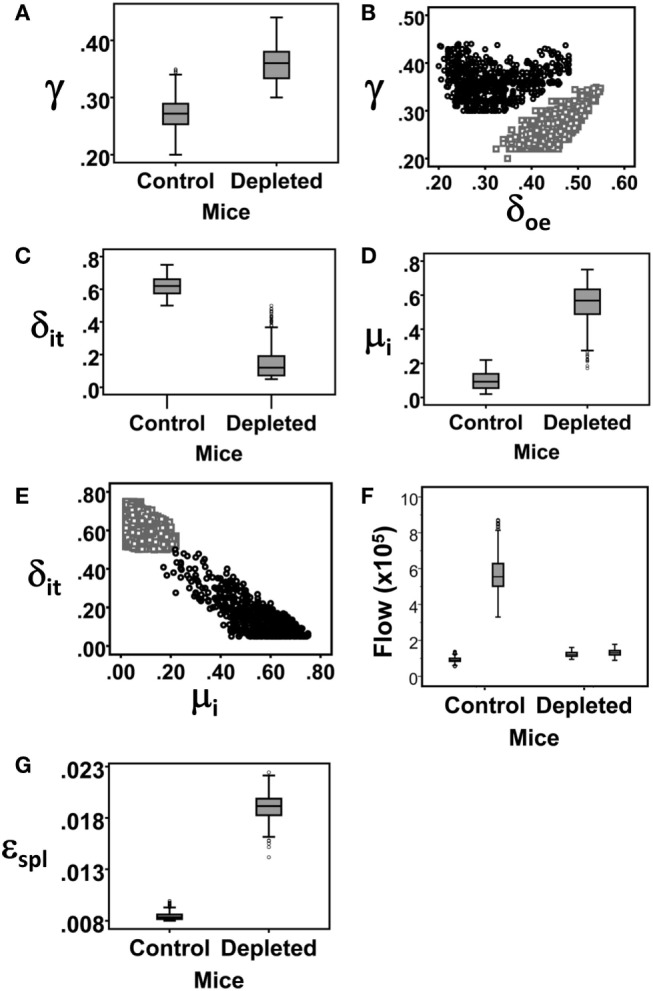
**Box Plots of the parameter value distributions for each rate parameter that was found to significantly differ between the simulations of control and depleted mice**. **(A)** The proliferation rate of the *B*_oe_ subpopulation. **(B)** The proliferation rate of the *B*_oe_ subpopulation plotted against its differentiation rate. **(C)** The rate of exit of immature B cell from the BM. **(D)** Immature B cell death rate. **(E)** The rate of exit of immature B cell from the BM plotted against their death rate. **(F)** Box Plots of the sources of mature recirculating B cell subpopulations, that is, the total cell number entering in each time step of 6 h. The white boxes are the flows from the splenic mature B cell subpopulation (ϕ_s_ × *B*_Mspl_) and the gray boxes are the flows from the BM immature B cell subpopulation (δ_i_re_ × *B*_i_). **(G)** The exit rate from the splenic mature B cell subpopulation.

**Figure 6:**
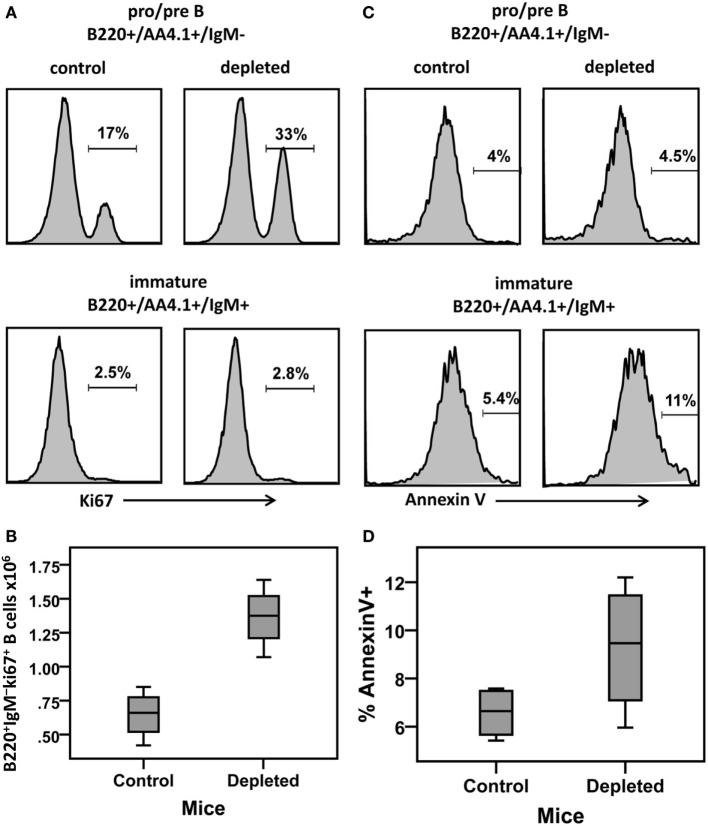
**BM cells from control and hCD20-depleted mice (34 days after depletion) were stained for B220 IgM AA4.1 and Ki67 or Annexin V**. For analysis, viable lymphocytes were defined by forward and side light scatter and the relative Ki67 or Annexin V staining in pro-/pre-B (B220^+^/AA4.1^+^/IgM^−^) or immature (B220^+^/AA4.1^+^/IgM^+^) B cell subsets was determined using gates set as shown in Figure 2A. **(A)** Histograms showing Ki67 expression in the gated pro-/pre- and immature B cell subsets in the control and depleted mice (representative example). **(B)** Ki67^+^ cell numbers in the pro-/pre-B cell compartment in control and B cell-depleted mice. **(C)** Histograms showing Annexin V expression in the pro-/pre-B and immature cell subsets in control and depleted mice (representative of four mice in each group). **(D)** Percentages of Annexin V^+^ cells in the pro-/pre-B and immature B cell compartments in control and B cell-depleted mice.

The *B*_oe_ subpopulation described above represents the pro- and pre-B cell populations, that is, the proliferating subsets which differentiate into immature B cells. Our simulation results reveal that the proliferation rate of the *B*_oe_ subpopulation in depleted mice is higher than the same rate in the control mice (Table 1; Figure 5A). This difference is not significant as the CI overlap. However, when we examine the interaction between the proliferation rate and the differentiation from this subset, we see that the parameters for each population form a different cluster, such that for all values of the other parameters, the depleted mice always have a higher proliferation rate with a lower differentiation rate than the control mice (Figure 5B). A lower differentiation effectively increases the cell residence time in the proliferating subset, thus further increasing the overall cell production. We validated this result by staining mouse BM cells with ki67, a cellular protein marker for proliferation, in the pro-/pre-B compartment in young control and B-cell-depleted mice (Figure 6A). The Ki67^+^ cell numbers in the depleted mice were significantly higher than those in the control mice (1.4 × 10^6^ ± 0.23 × 10^6^ and 0.65 × 10^6^ ± 0.18 × 10^6^, respectively; *p* = 0.003) (Figure 6B). This result supports the model’s prediction that reconstitution of BM B cell numbers after a short depletion requires an increased proliferation of these cells.

### Accelerated Immature B Cell Death and Lower Differentiation Rates in Depleted Mice

The most significant changes were observed in the immature cell death rate and their differentiation rate into transitional B cells (Table 1). In control mice, the majority of cells left the immature compartment by differentiating into transitional B cells and fewer by death. On the contrary, in the depleted mice, most of the immature cells left this subset by dying, and fewer cells transited to the spleen (Table 1; Figures 5C–E). The differentiation rate of immature B cells into the BM mature B cell subset and the rate of their reflux back to the pro-/pre-B cell compartment were the same in control and depleted mice. Differentiation and death thus balanced each other, so that the residence time [defined as 1/(death rate + exit rate)] in the immature B cell subpopulation was the same in both control and depleted mice (Table 1). To validate the increased immature B cell death in the depleted mice, we stained mouse BM cells with Annexin V, which identifies pro-apoptotic cells, and quantified this staining within the gated immature B cell subset (B220^+^/AA4.1^+^/IgM^+^) in young controls and B cell-depleted mice (Figure 6C). The frequency of Annexin V-positive cells among immature B cells from depleted mice was twofold larger relative to immature B cells from control mice, whereas no difference in apoptosis was found in the pro-/pre-B cell compartment (Figure 6D). This result supports the model’s prediction that apoptosis is increased in immature B cells in depleted mice.

### Mature Recirculating B Cells in the BM Probably Come from the Periphery

There were no significant differences in the input into, and the exit rate from, the mature recirculating B cell subpopulation in the BM marrow of depleted vs. control mice (Table 1). However, this is the first time that this subpopulation is included in a mathematical model, so it was of interest to examine its kinetic parameters. The source of the BM mature B cell subset is uncertain, as we cannot distinguish experimentally between naïve mature cells that have developed from the immature B cells in the BM and cells that have emigrated from the periphery into the BM. Our model predicts, however, that the most probable source of this subpopulation in control mice is inflow from the splenic mature subpopulation (3.3–8.7 × 10^5^ cells/6 h), while the extent of direct differentiation is almost an order of magnitude lower (5.5 × 10^4^–1.4 × 10^5^ cells/6 h).

The flow rates from the splenic B cells to the BM were the same in the control and depleted mice; however, the total number of splenic mature cells that flow back into the BM is lower in the latter mice due to the depletion (Figure 5F). Hence, the total numbers of mature recirculating B cells are lower in the depleted mice compared to the controls, in both the experimental data and the model simulation (Figure 4B). Finally, the exit rate from the splenic mature B cell subpopulation was higher in the depleted compare to control mice (Table 1; Figure 5G), possibly due to demand from other peripheral lymphoid tissues.

### Parameter Sensitivity Analysis

We used linear regression analysis to determine which parameters are critical for the model outcome, that is, the total cell numbers in each subpopulation. Along with the kinetic rates, we included the mouse type (control or depleted) as a parameter. The critical parameters determining immature cell numbers were found to be mouse type, all the exit rates from this subpopulation and the proliferation rate of the pro-/pre-B subpopulation (Table 2). These parameters together explain 98% of the variance in immature total cell numbers (*R*^2^ = 0.98). Increasing the pro-/pre-B proliferation rate (positive partial correlation) or decreasing any of the immature B cell exit rates (negative partial correlation) would increase the total cell numbers in the immature compartment (Table 2), as expected. The positive partial correlation between mouse type and immature B cell numbers suggests that the depletion results in slightly increased cell numbers in the immature B cell compartment.

**Table 2:** **Sensitivity analysis by linear regression**.

Model outcome	*R*	*R*^2^	Critical parameters	Partial *R*
Immature cell numbers (*B*_i_)	0.99	0.98	Mouse type	0.81
			δ_i_re_	−0.77
			μ_i_	−0.90
			δ_i_t_	−0.89
			γ	0.61

Transitional cell numbers	0.98	0.97	δ_i_t_	0.69
(*B*_t_)			γ	0.50
			μ_i_	−0.62
			δ_i_re_	−0.26
			μ_t_	−0.62
			δ_t_	−0.59

BM recirculating mature	0.97	0.95	Mouse type	−0.88
cell numbers (*B*_Mrec_)			δ_i_re_	0.74
			γ	0.59
			ϕ_BM_	−0.54
			ϕ_s_	0.47

Splenic mature cell	0.98	0.96	δ_t_	0.40
numbers (*B*_Mspl_)			μ_i_	−0.81
			μ_t_	−0.53

The critical parameters affecting transitional B cell numbers were, as expected, the rate of immature B cell differentiation into this subset, all the other exit rates from the immature B cell subset (as these processes compete with exit to the periphery), the exit rate from this subpopulation, and the proliferation rate of the pro-/pre-B subpopulation, through its effect on immature B cell numbers (Table 2). These parameters together explain 97% of the variance in transitional B cell numbers. Increasing pro-/pre-B proliferation and the immature to transitional differentiation rate, or decreasing any of the other exit rates from the immature and transitional subpopulations, would increase the cell numbers in the transitional compartment.

Cell numbers in the BM mature recirculating B cell subset were similarly affected by mouse type, the immature B cell rate of differentiation into this subset, the proliferation rate of the pro-/pre-B subpopulation, the inflow from the periphery into this compartment, and the flow out of this subpopulation to the periphery (Table 2). These parameters together explain 95% of the variance in BM mature B cell numbers. When the rates of pro-/pre-B proliferation, immature B cell differentiation or inflow from the periphery increase, or the flow of mature B cells from the BM to the periphery decreases, the BM mature B cell numbers increase. The negative partial correlation between mouse type and BM mature B cell numbers suggests that the depletion slightly decreases these cell numbers.

Finally, the critical parameters affecting splenic mature B cell numbers are the rate of transitional cell maturation to this subset and the death rates of both the immature and transitional B cell subpopulations (Table 2). These parameters together explain 96% of the variance in splenic mature B cell numbers. When transitional cell maturation increases, or any of these death rates decreases, the splenic mature B cell numbers increase, as expected.

## Discussion

Cellular homeostasis of the peripheral B cell compartment in the BM is maintained by a balance between cell production and cell death (13). In the present study, we have tested the previously suggested (14, 15), but yet not proven concept that, similar to other hematopoietic cell lineages (46–48), B lymphopoiesis is regulated by the size of the peripheral B cell compartment. We have used the human CD20Tg mouse model, where peripheral B cells can be specifically eliminated using anti-CD20 antibodies with no residual effect on other cell populations, in order to find whether mature B cells affect the homeostasis of developing B cells in the BM (25). We used flow cytometric analysis, BrdU labeling, and our mathematical models of B cells development and maturation, to compare between B cell population dynamics in short-term (34 days) depleted mice and in control mice.

The mathematical model results suggested that in depleted mice, pro-/pre-B cells have a higher proliferation rate and a lower differentiation rate, relative to control mice (Table 1; Figures 5A,B). Since the effect of lowering the differentiation rate is to increase the residence time of B cells in this compartment, thus giving them more opportunities to divide, the net effect of these two changes is to increase overall B cell production. This finding was validated by ki67 staining (Figure 6), supporting the conclusion that depletion of transitional and mature B cells results in increased B cell production in the BM. Importantly, we showed above that the pro-/pre-B cells in this mouse model do not express hCD20, and therefore were not affected by the anti-hCD20 antibody. This suggests that the increased proliferative response we found in this cell population is driven by the alteration of B cell homeostasis caused by the selective depletion of immature and peripheral B cells and not due to a general elimination of all B cell subsets.

To characterize homeostatic effects, they must be measured upon alteration of homeostasis under highly physiological conditions. We believe the hCD20Tg model is most appropriate, as it allows selective depletion only of peripheral mature B cells with no residual effects on other cell lineages (25). Studies with other lymphopenic mouse models, such as BAFF-receptor mutants, CD19^−/−^ and CD74^−/−^, revealed no apparent effect on B cell generation (49–51). However, in these mice, B cell generation was determined based on quantification of B cell subsets at a single time point, rather than by kinetic measurements that would enable the calculation of proliferation and death rates, as we have done here. Nevertheless, we have recently shown that B cell lymphopenia in these mouse models prevents the age-associated decline of B lymphopoiesis that is otherwise developing in aged normal mice (32, 34), and that B cell depletion in normal old mice reactivates B lymphopoiesis in the BM (32). These findings, in our view, strongly support the existence of a feedback mechanism by which the peripheral B cell compartment regulates B lymphopoiesis. The nature of this homeostatic regulation is yet elusive. It is tempting to speculate that a soluble factor that is secreted by peripheral B cells, or other cells that somehow sense B cells numbers, can suppress/stimulate B lymphopoiesis in the BM, similarly to the role of erythropoietin in maintaining red blood cell homeostasis. However, simpler mechanisms may exist, such as competition for BM survival niches or contact-mediated effects, as we have proposed (52–54). Further studies are needed in order to identify this mechanism.

The current model did not address the question of whether homeostatic regulatory mechanisms also affect the entry of hemopoietic stem cells into the pro-/pre-B cell compartment for two reasons. First, the experimental data did not cover these subsets and second, the effect of increased entry into this compartment on subsequent compartments would be similar to that of increased proliferation. Once the nature of the homeostatic regulation mechanism is elucidated, however, it would be interesting to examine its effect, if any, on stem cell differentiation into the B cell lineage.

The model also did not address the possibility that any other cell subsets affect the dynamics of B cell development in the BM, because the depletion only affects the B cell compartment (25), and thus, we could not use the depletion experiments to elucidate the effects of other cell subsets. However, our results enable us to exclude the possibility of competition for BM survival niches by other cells – such as plasma cells, which might have been indirectly affected by B cell depletion – because such competition would have changed *K*, the carrying capacity limiting the proliferation in the pro-/pre-B cell compartment. However, no such difference in *K* between control and depleted mice was obtained in fitting the model to the experimental data.

In addition to the effects on the pro-/pre-B cell compartment, the modeling results revealed strong effects of depletion on the immature subset. These include both an accelerated immature B cell death and a lower rate of differentiation of immature to transitional B cells in depleted mice compared to the rates in control mice, although the residence times in these compartments were the same in both control and depleted mice (Table 1; Figures 5C–E). As a result, the total cell numbers of the more developed stages are lower in depleted mice. As the increased immature cell death during recovery from depletion was surprising, we validated this result experimentally. The frequency of Annexin V-positive cells among immature B cells in depleted mice was twofold larger than that in control mice (Figure 6D), validating the model’s prediction. It is plausible that when cells numbers are reduced by depletion, there is less competition for binding self-antigens, so that negative selection pressures may increase; if true, this would explain the increased death and reduced differentiation of immature B cells.

Finally, our modeling results predict that the source of the mature B cells in the BM is probably an inflow from the periphery to the BM, and that it is less likely that this population develops directly from the immature B cells subpopulation in the BM. Thus, the mature B cells in the BM are, indeed, recirculating cells and not BM-resident. In situations where peripheral B cells numbers increase – as in aging – then this predicts that mature B cell recirculation to the BM, and homeostatic pressures on developing B cells in the BM, will also increase.

Our results suggest that the homeostatic pressures exerted by increased numbers of peripheral B cells, as in lymphomas, in autoimmune diseases where the B cell population includes expanded self-antigen-specific B cell clones, or in the elderly where peripheral B cells include high numbers of long-lived antigen experienced B cells, probably affect B cell production in all these situations. Rituximab, a chimeric monoclonal antibody directed toward the pan-B cell surface marker CD20, is widely used in the treatment of B cell malignancies and autoimmune diseases, with very promising results (55). In this study, we simulated the Rituximab B cell depletion treatment and defined its effect on B cell production dynamics. The increased B cell production observed in depleted mice indicates that, indeed, the BM mature B cells are being replaced during post-depletion recovery by a variety of newly generated cells. This may contribute to better immune recovery in those patients in whom treatment has been completed and the B cell compartment regenerated, as the new repertoire would be “rejuvenated” by the treatment.

## Author Contributions

DM and RM designed the study. SZ-R performed most of the experiments and DB performed some experiments. GS performed all the modeling, statistical analysis, and wrote the paper. DM and RM critically read and finalized the paper.

## Conflict of Interest Statement

The authors declare that the research was conducted in the absence of any commercial or financial relationships that could be construed as a potential conflict of interest.

## Abbreviations

AIC, Akaike’s information criterion; BCR, B cell antigen receptor; BM, bone marrow; LHS, Latin hypercube sampling; MLE, maximum likelihood estimator.
